# Preparation and Characterization of Polyanhydride Terminated with Oleic Acid Extracted from Olive Mills Waste

**DOI:** 10.3390/polym14224799

**Published:** 2022-11-08

**Authors:** Mustafa Zakiedin, Mansour Alhoshan, Maher M. Alrashed, Lahssen El Blidi

**Affiliations:** Department of Chemical Engineering, King Saud University, Riyadh 11421, Saudi Arabia

**Keywords:** oleic acid, melt polycondensation, terminated polyanhydrides, olive waste, sebacic acid

## Abstract

Valorizing the fatty content of agricultural waste in material synthesis is an interesting topic. This work focused on utilizing oleic acid from the solid waste of olive mills in Saudi Arabia to synthesize biodegradable polyanhydrides based on sebacic acid which terminated with different concentrations of fatty acid (10, 30, 50, and 70 wt%), then characterize the final polymer samples and study the effects of termination on polyanhydrides properties, such as molecular weight and degradation profile. The fatty content of the solid waste was extracted, purified, and analyzed prior to and after separating the saturated and unsaturated fractions by urea crystallization, then the microwave-assisted melt polycondensation technique was used in the synthesis of the final polymers. Molecular weights were determined by gel permeation chromatography (GPC), and the degradation profile of the prepared samples was examined by determining the weight loss percentage of the polymer mass and FT-IR scanning for the anhydride bond before and after sample degradation. Results showed a linear degradation profile for most samples with no significant change in the molecular weights due to termination.

## 1. Introduction

**Figure 1:**
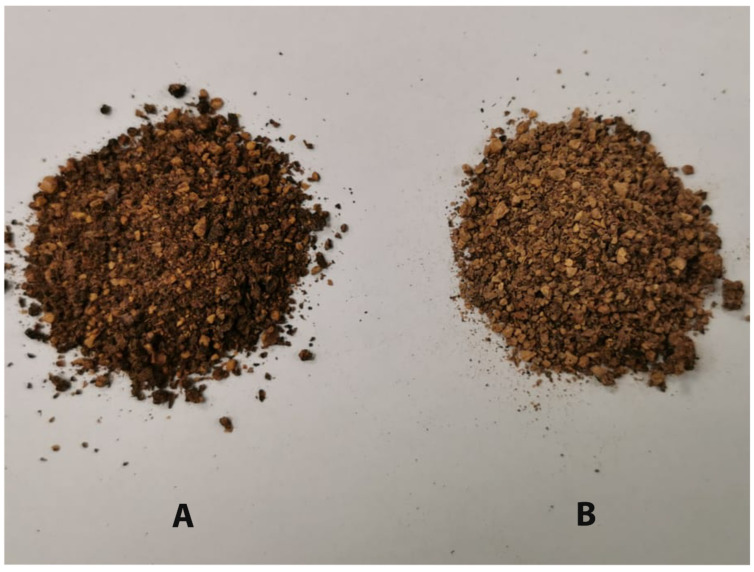
Solid waste of olive milling process (pomace), (**A**) before solvent extraction. (**B**) After solvent extraction.

In a recent official report, it was outlined that almost all the olive trees in the Kingdom of Saudi Arabia are located in the Al-Jouf area (northern region) which produces over 278,000 tons of olive oil annually [1]. The numbers on the same report reflect that olive trees in KSA are the most highly planted evergreen trees aside from date palm, with over 14 million trees, and about 65% of all olive trees are productive or fruitful. Most, if not all, of this cultivation is used to produce virgin olive oil in both traditional and modernized industrial-scale olive mills. The process of producing raw olive oil generates bulk amounts of moist solid waste (pomace) shown in Figure 1. This waste is loaded with an appreciable amount of remaining oil, moisture and other organic active materials, such as sugars and polyphenols depending on the efficiency of the milling process. Although farmers tend to sell these pomaces for oil refineries to produce second-grade pomace olive oils, a considerable amount of it ends up either burned close to the mills or dumped on large landfills, which generates environmental issues. A recent review found that oily solid wastes which cover the riverbed eventually result in the death of the most sensitive organisms [2]. Polyanhydrides are a unique class of biodegradable polymers because of the two hydrolysable sites in their repeating unit which are connected by the anhydride bonds; this feature has subjected the polyanhydrides to extensive research and studies as biopolymers for controlled release applications [3,4,5]. In vitro and in vivo studies of polyanhydrides biodegradability have proven that these bio-erodible polymers can degrade into non-cytotoxic components [6,7]. Based on the chemistry of the monomer units and the degree of hydrophilicity or hydrophobicity, polyanhydrides can be divided into aliphatic, aromatic, unsaturated, and aliphatic–aromatic homo-polyanhydrides called conventional polyanhydrides. Advanced polyanhydrides include cross-linked polyanhydrides, poly(ester-anhydride) polymers, fatty acid-based polyanhydrides, amino acid-based polyanhydrides, poly(anhydride-co-imides), polyanhydrides with polyethylene glycol functionality [3]. To improve the flexibility, low melting temperature, hydrophobicity, and pliability of polyanhydrides, fatty acids were incorporated as monomers using carboxylic acid functionality. This is done without reducing the overall biodegradability of the polymer because fatty acids degrade into naturally occurring compounds, making them environmentally friendly. The main role of mono-functional fatty acids was to act as a chain terminator in the polymerization process [8]. Unsaturated fatty acids can only be polymerized after being converted to a bifunctional monomer by dimerization or creating an additional functional group on the fatty acid. Fatty acid-based polyanhydrides are typically hydrophobic, non-, or semi-crystalline, soluble in most chlorinated organic solvents and have a low melting point range of (20–90 °C) with low mechanical strength. A ricinoleic acid (RA)-based polymer, such as RA (cis-12-hydroxyoctadeca-9-eonoic acid), is most suitable for the synthesis of the fatty acid-based polyanhydrides because it is bifunctional and possesses an additional 12-hydroxy group alongside the acid group [8,9]. Polyanhydrides with hydrophobic terminals, such as sebacic acid (SA) were melt-polymerized in excess acetic anhydride to form the prepolymer of poly(sebacic anhydride) (PSA), In this scheme, the hydrophobicity is increased by interchanging the polyanhydride chain with the prepolymer of the fatty acid anhydrides of acetic acid [10].

This work suggests utilizing the fatty acid content of olive pomace in the synthesis of fatty-acid-terminated-polyanhydride based on oleic acid and sebacic acid with different ratios; then studying and analyzing the synthesized polymer characteristics, such as degradability, and molecular weights. The prime motivation behind this work is the lack of research about fatty-acid-terminated polyanhydrides and the promising approach of valorizing olive solid waste in the advanced materials field.

## 2. **Materials and Methods**

### 2.1. Materials

Urea (purity ≥ 99%, Bio Basic, Inc.), oleic acid (Acidimetric 197–202 mg, Fisher${}^{®}$ Chemical), acetic anhydride, AR (purity ≥ 98%, AVONCHEM${}^{®}$), buffer solution (pH7.0 (20 °C), Certipur${}^{®}$), sebacic acid (purity ≥ 99.5%, SAOC${}^{®}$) diethyl ether (purity ≥ 99.8%, Sigma Aldrich${}^{®}$), n-hexane (purity ≥ 99% (GC), Honeywell${}^{®}$), petroleum ether (purity ≥ 95%, Sigma Aldrich${}^{®}$), methanol (purity ≥ 99.8% (GC), Sigma Aldrich${}^{®}$), tetrahydrofuran (purity > 99.5%, Fisher${}^{®}$ Chemical), hydrochloric acid 37% (Acidimetric 36.5–38.0%, Panreac${}^{®}$), acetone (purity ≥ 99.5%, Sigma Aldrich${}^{®}$), sodium sulfate anhydrous ( purity > 99%, Fisher${}^{®}$ Chemical), ethanol (purity > 99.8%, CHEM-LAB${}^{®}$), sodium hydroxide (Acidimetric ≥ 98%, Panreac${}^{®}$) and potassium hydroxide (purity as KOH ≥ 85%, Loba Chemie${}^{®}$) all were used as supplied. Deionized water was obtained by instrument (Milli-Q${}^{®}$ Direct ultrapure water, Millipore${}^{®}$ System). Olive solid waste samples were provided by local farmers and mills in the northern region of Saudi Arabia.

### 2.2. Oil Extraction from Olive Waste

**Figure 2:**
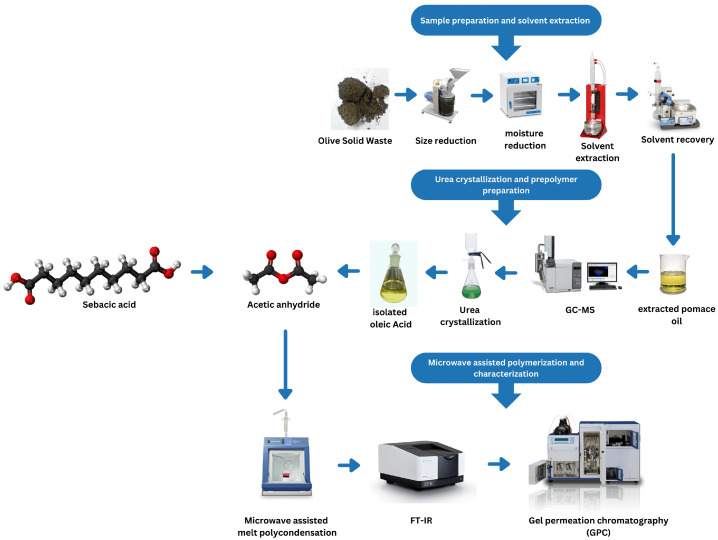
Schematic illustration of the experimental steps.

Figure 2 below demonstrate the followed experimental sequence. Firstly, the drying process aimed to reduce the moisture content of the olive pomace samples and help to break the outer surface of the olive pomace, which allows the solvent to dissolve more easily. The samples were finely ground to a size of (0.2–1 mm), then $M_{initial}$ was taken, then dried overnight using a drying oven at a temperature of (70–105 °C). The samples were weighed again the next day, and the $M_{Dry}$ and $Moisture\;\left(\%\right)$ were calculated by the following equation:

$$\begin{array}{c} Moisture\;\left(\%\right)=\;\frac{M_{initial}-\;M_{Dry}}{M_{initial}} \end{array}$$

#### 2.2.1. Pretreatment of Waste with Sodium Chloride

To enhance the extraction rate later in the Soxhlet, the dried solid samples were soaked in a sodium chloride solution (2 M) for 24 h then rinsed with distilled water and dried for 3 h at 70 °C. Selecting the optimal concentration of the NaOH solution will facilitate the rupture of olive cake cells, which increases the oil extraction process. The concentration of (2 M) was used based on previously reported work, which was made on the same type of waste [11].

#### 2.2.2. Pomace Oil Extraction and Solvent Recovery

The treated pomace samples (2255 g) were loaded into the Soxhlet extractor (205 g, each run), then hexane (0.5–1 L) was added to the bottom flask. The condenser line was connected, and the heating mantle was turned on. The temperature was controlled at 70 °C for 8–10 h. At the end of the extraction, the system was turned off and the oil–solvent mixture was left to cool. By using a rotary evaporator, the solvent was recovered and kept in a separate bottle. Finally, the extracted pomace oil was measured, and the weight was recorded. The percentage yield of the extracted oil (Y%) was calculated to be 17% using the following equation:

$$\begin{array}{c} Y\%=\;\frac{M_{2}}{M_{1}}\times 100 \end{array}$$

where *M*${}_{2}$ is the weight of the olive oil extracted and *M*${}_{1}$ is the initial weight of the olive waste.

#### 2.2.3. Determining Acid Value and Free Fatty Acids (FFA%)

The acid value was determined quantitatively using the titration method. First, a phenolphthalein indicator was prepared by mixing and dissolving 2 g of phenolphthalein powder with 100 mL of ethanol. Then titrant solution of 0.1 N sodium hydroxide was prepared by dissolving 4 g NaOH pellets into 900 mL of distilled water under temperature of 60 °C. The mixture was later cooled, and distilled water was added to make up the final volume of 1 L.

Then, 50 mL of ethanol was measured in a conical flask, and 2–3 drops of phenolphthalein indicator was added. The mixture was neutralized by adding the titrant solution from a burette until a faint purple color appeared. The initial reading was recorded, then 10 mL of the oil sample was measured and mixed with the neutralized mixture. Finally, 3 drops of the indicator was added, and the mixture was titrated until the end point.

The acid value (oleic acid) was calculated to be 1.68 mg (KOH)/g (oil) using the following formula:

$$\begin{array}{c} ACID\;VALUE\;\left(Oleic\;Acid\right)=\;\frac{56.1\;\times V\times N}{W} \end{array}$$

where 56.1 = Potassium hydroxide molecular weight (g/mol) and *V* = titrant solution volume = final burette reading − initial burette reading (mL). *N* = The normality of titrant solution = 0.1 N. *W* = the oil sample weight (g).

Similarly, the free fatty acids (FFA%) was calculated to be 0.84 % by the weight of the sample. The following formula was used:

$$\begin{array}{c} FFA\;\%\;\left(Oleic\;Acid\right)=\;\frac{ACID\;VALUE\;(Oleic\;Acid)}{2} \end{array}$$

### 2.3. Oleic Acid Separation and Purification

#### 2.3.1. Transesterification

A total of 200 g of oil was reacted with a solution of 44 g methanol and 2 g potassium hydroxide (KOH). The reactant mixture was stirred under temperature of (65–70 °C) for 60 min. After the reaction was complete, the mixture was transferred into a separation funnel and left to settle. Two distinct layers were observed: an upper layer rich with fatty acid methyl esters (FAME) and a lower layer of glycerol. After the separation of the glycerol (40 mL), the upper layer of FAME was washed with HCL (50 mL) and distilled water (100 mL) to adjust the pH before dissolving in hexane (150 mL) to separate any remaining water. The hexane-FAME mixture was transferred to the rotary evaporator to recover the solvent and finally dried by sodium sulfate. The final obtained weight of FAME was 175.5 g with a % yield of 88.

#### 2.3.2. Urea Extraction Crystallization

**Figure 3:**
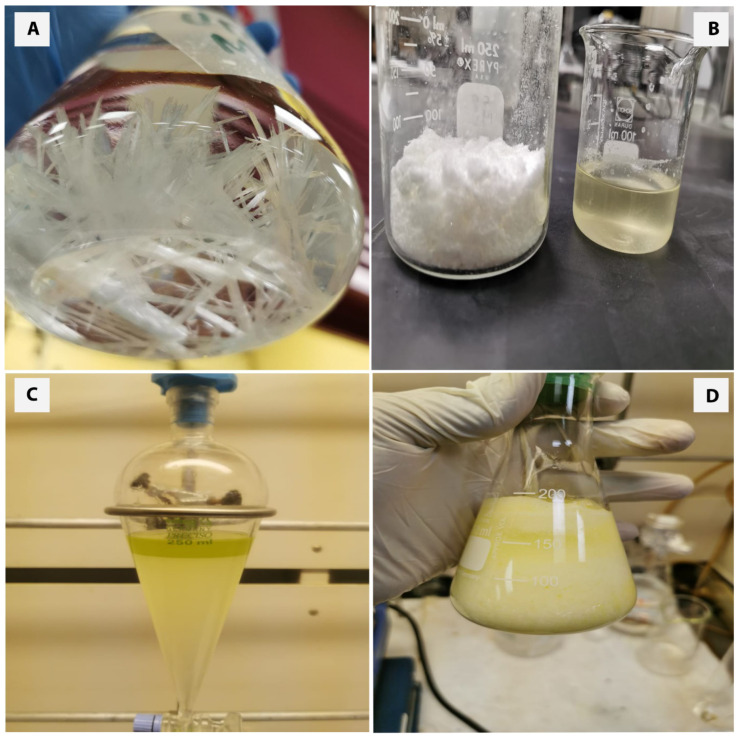
(**A**) Urea solution crystallization when temperature decreases. (**B**) Urea–methanol mixture after vacuum filtration. (**C**) washing with HCL-water solution and separation into upper layer of fatty acids and lower layer of urea concentrate. (**D**) Homogeneous mixture of urea, methanol, and fatty acids after cooling.

**Figure 4:**
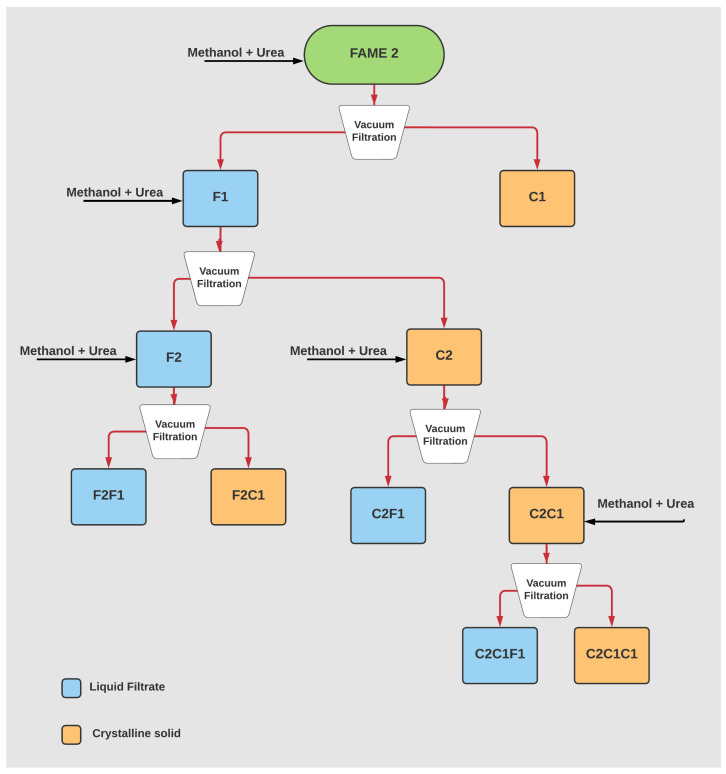
Urea crystallization sequence illustration.

A total of 100 g of urea was dissolved in 500 mL methanol (Figure 3A) under reflux at 70 °C, then 100 g of the FAME was added slowly until a homogeneous mixture was formed (Figure 3D). Next, the mixture was left to freeze overnight at a temperature range of (−5 °C to −10 °C) [12,13]. The mixture was then filtered at room temperature. Two phases resulted from the filtration: a white crystalline powder (C1) and liquid filtrate (F1) (Figure 3B). (C1) was washed with the HCL-water solution and separated in the separation funnel into an upper layer of fatty acids and lower layer of urea concentrate as demonstrated in Figure 3C. The upper layer was separated and dissolved in 150 mL of hexane before being transferred into the rotary evaporator, then it analyzed by GC/MS to determine the fatty acids’ profile. A new extractions crystallization was conducted on the liquid filtrate (F1), which resulted in a white crystalline powder (C2) and liquid filtrate (F2). Following the same sequence, three more crystallizations were performed as shown in Figure 4. After each followed filtration, the solid and liquid phases were similarly washed with the HCL-water solution and separated in the same way before being analyzed by GC/MS.

### 2.4. Polyanhydride Synthesis

#### 2.4.1. Prepolymer Synthesis

The sebacic acid diacid monomer was first purified by refluxing it in excess acetic anhydride for 30 min, and the solvent was evaporated to dryness. The formed clear viscous residue was filtered, dissolved, and left to precipitate in a mixture of ether/petroleum ether (1:1 *v*/*v*). Finally, a white precipitate was formed, filtered, and dried at room temperature. The oleic acetate anhydride was prepared by dissolving oleic acid in acetic anhydride by a ratio of 1:5 *w*/*v*, then the mixture was refluxed for 30 min at 150 °C and evaporated to dryness. The followed procedure of preparing the prepolymers was previously reported for various types of fatty acids [14]. For polyanhydrides which terminated by waste oleic acid, only sample C2C1F1, resulted from the above urea crystallization, was used because it contained the highest oleic concentration.

#### 2.4.2. Polymerization

Different concentrations of sebacic acid prepolymer and oleic acetate anhydride were reacted in the microwave at 150–160 °C for 5–10 min [5,15]. Due to the difficulties of mixing in conventional microwaves, the reactant flask was mixed manually, and the temperature was measured on each time set. Samples which were abbreviated by (STD) were synthesized by standard purity oleic acid, e.g., STD 90:10 represents polyanhydride consisting of 90% weight of sebacic acid and 10% weight of standard oleic acid. In contrast, samples which were abbreviated by (SYN) were synthesized by oleic acid, which was extracted from waste olive oil.

### 2.5. Characterization

#### 2.5.1. GC-MS

Gas chromatography was performed with a setup having the following characteristics: name: SHIMADZU QP2010 Ultra; column oven temp: 115.0 °C; injection temp: 225.00 °C; injection mode: split flow control mode; pressure: 100.0 kPa; total flow: 50.0 mL/min; column flow: 3.36 mL/min; linear velocity: 68.6 cm/s; purge flow: 3.0 mL/min; split ratio: −1.0.

#### 2.5.2. FT-IR Analysis

Samples were scanned by SHIMADZU IRPrestige-21 equipped with a bright ceramic light source and signal-to-noise ratio, 40,000:1 and 98% internal mirror reflectance.

#### 2.5.3. Gel Permeation Chromatography (GPC)

The analysis parameters were as follows: Viscotek TDA 305/solvent = THF; Column set = 1 − PLGel 5 μm + 5 μm Guard; sampling flow rate = 1 mL/min; injection volume = 100 μL; detector temp = 35 °C; column temp = 35 °C.

#### 2.5.4. Degradation Analysis

**Figure 5:**
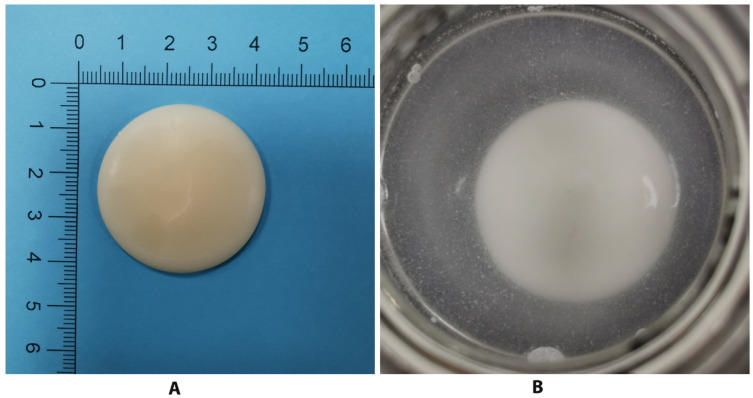
(**A**) Final polyanhydride sample after being melt-casted (4 mm thick) at 80 °C. (**B**) The polymer submersed in buffer solution (PH: 7.0).

Samples of polymers were prepared and measured (3 g of each) then placed in a round glass mold and melt-casted (4 mm thick) at 80 °C (Figure 5A). All samples were left to cure and dry for 2 days before the degradation experiment. The samples were then submersed in a 30 mL phosphate buffer solution of pH (7.0) and placed in the incubator (100 RPM at 37 °C) (Figure 5B). At each time set, the samples were taken out from the incubator, rinsed, dried and weighted, then new amounts of phosphate buffer solution were added. The experiment was held for 42 days, and the percentage mass loss of each polymer was recorded. The % mass loss (wt%) was calculated by the following formula and plotted for each sample [16]:

$$\begin{array}{c} wt\%=\frac{\left[initial\;sample\;weight\left(g\right)\right]-\left[dried\;sample\;weight\left(g\right)\right]}{\left[initial\;sample\;weight(g)\right]}\times 100 \end{array}$$

## 3. Results and Discussion

### 3.1. Fatty Acid Methyl Esters (FAME) Characterization

**Figure 6:**
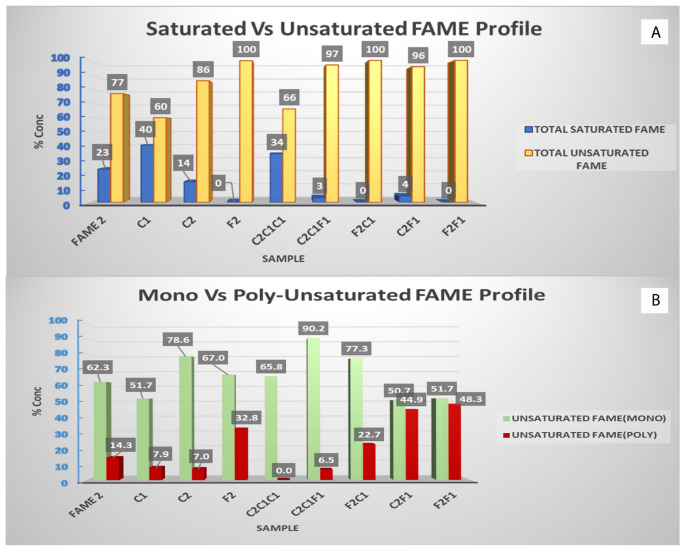
(**A**) Saturated vs. unsaturated methyl esters content on samples after urea crystallization. (**B**) Mono vs. poly-unsaturated methyl esters content on samples after urea crystallization.

**Figure 7:**
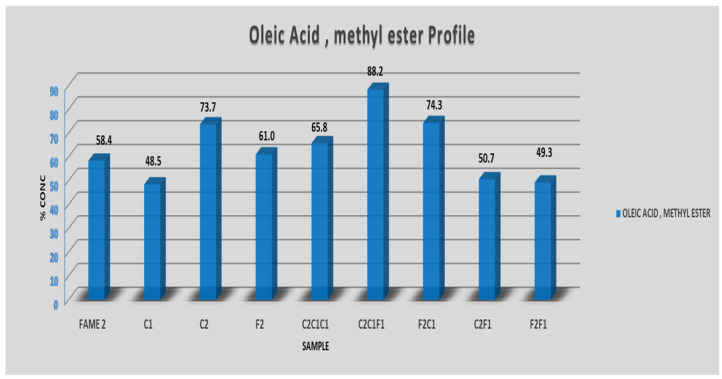
Concentration of oleic acid on each sample.

Following the urea separations, the first step of characterizing the samples is to check the fatty acid methyl esters (FAME) profile after each separation. Samples were scanned by GC/MS, and Figure 6A shows the saturated vs. unsaturated FAME profile of all filtrates and solids left behind. The initial oil sample (FAME 2) contained 77% of the total unsaturated fatty acid methyl ester (FAME); this includes both mono- and poly-unsaturated FAME. In contrast, the total saturated FAME in the starting sample was 23%; this increase in unsaturated FAME is thought to be conventional in most olive oil grades and types. After the first filtration, most of the saturated FAME was extracted, and the unsaturated FAME was found to be dominant in the following samples. Nearly in all liquid filtrate samples or solid crystals of filtrate (F2, F2C1 and F2F1), the unsaturated FAME reached 100% concentration. This confirmed that the kink in the fatty acids chain which was caused by the unsaturation bond (mono or poly) facilitated their separation based on size and prevented the straight chain saturated FAME from coexisting in the filtrate; this for sure depends on how perfect the 5.67 Å diameter of urea crystals is formed during the temperature decrease. In Figure 6B, which presents the mono- and poly-unsaturated FAME, a clear pattern of increased polyunsaturated (more than one kink in chain) was observed in the solid phase; for example, in sample C2, the polyunsaturated FAME represented 7% of the total unsaturated fraction while in sample F2, which is the liquid filtrate, the percentage increased to 32.8%. This gives another indication of urea effectiveness in separating FAME based on their saturation degree. Accordingly, Cis-oleic acid (C18:1), being mono-unsaturated, was expected to be found in higher concentrations in liquid filtrates. The highest concentration of oleic acid was 88% and was achieved in sample C2C1F1 after five filtrations (Figure 7). A visible indication was observed in the samples with high oleic acid concentration, which are colorless to slightly yellowish.

The concentration difference of saturated and unsaturated fatty acids before and after the urea crystallization is evident by comparing the chromatogram of the initial sample FAME2 and sample C2C1F1 (Figures S1 and S2), which shows the highest oleate concentration. A clear reduction in saturated palmitate (C16:0) and stearate (C18:0) was noticed; likewise, the concentrations of unsaturated palmitoleate (C16:1) and lineoleate (C18:2) were also reduced after the purification.

### 3.2. Fourier-Transform Infrared Spectroscopy (FT-IR)

After polymerization, the samples were analyzed with FT-IR to confirm the formation of the anhydride bond and to examine the purity of the prepared samples. Figures S3 and S4 represent the FT-IR spectra of the polyanhydrides that were synthesized. All spectra were characterized by a strong absorption due to the carbonyl group of the carboxylic acid near (1700–1711 cm${}^{-1}$) which can be an indication of traces of unreacted acetylated prepolymer or acetic anhydride that remained in the prepolymer and did not fully evaporate before the final reaction in the microwave. It could be also a non-reacted oleic acid or sebacic acid, which is more unlikely in this case because typically, carbonyl bond from carboxylic acids usually accompany a hydroxyl (-OH) absorption band at a range of (3250–3650 cm${}^{-1}$) which was not observed in all samples [17]. Typically, the peaks representing the anhydride bonds appear as pairs (bimodal) because anhydrides exhibit two stretching bands in the carbonyl region, which result from symmetrical and asymmetrical carbonyl stretching modes. In the double bond region, two characteristic anhydride peaks could be identified at (1745–1815 cm${}^{-1}$), which represent the sebacic anhydride formation [8,14]. Strong intensity absorption in the range of (2850–2935 cm${}^{-1}$) was observed in all samples, and it mostly related to a C-H stretch of a long chain linear aliphatic part. No triple bond wavelength was observed at (2000–2500 cm${}^{-1}$). An overlay comparison of the fingerprint region (600–1500 cm${}^{-1}$) confirmed a semi-identical fingerprint for all samples. Typically, all reported fatty acid anhydride derivatives have typical IR absorption in 1740 and 1810 cm${}^{-1}$ (symmetrical and asymmetrical anhydride C=O stretching bands) with no carboxylic acid peak in 1700–1730 cm${}^{-1}$. ^1^H-NMR spectra of all prepolymers showed a typical singlet peak at 2.22 ppm attributed to the acetyl-terminated end group [10,14].

### 3.3. Molecular Weight Determination by Gel Permeation Chromatography (GPC)

**Figure 8:**
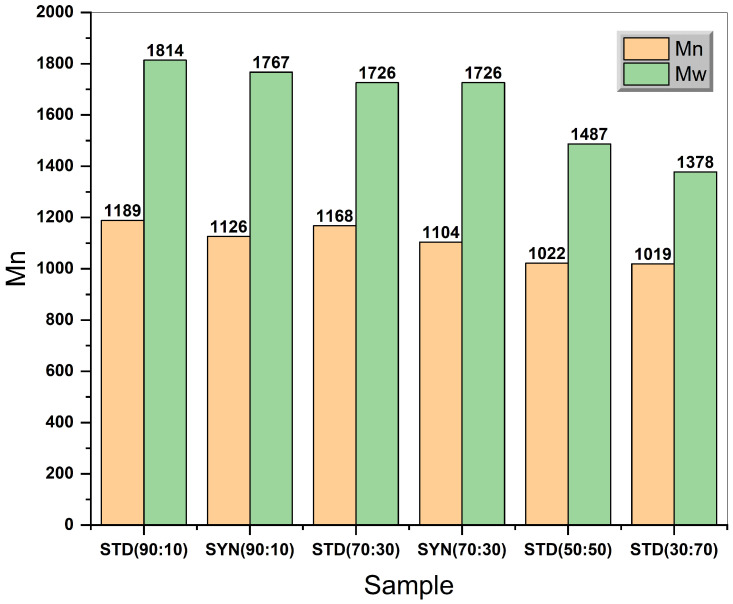
Representation of M${}_{n}$ and M${}_{w}$ of each sample.

The molecular weight profile indicates a close correlation between sebacic acid concentration and molecular weight in both number average molecular weight and weight average molecular weight as shown in Figure 8. A relevant decrease in both M${}_{n}$ and M${}_{w}$ was observed when the concentration of sebacic acid was less than or equal to 50 wt% as shown by samples STD (50:50) and STD (30:70). Samples terminated with standard grade oleic acid have slightly higher M${}_{n}$ than samples which were terminated from the olive waste extract. For instance, sample SYN (90:10) had M${}_{n}$ of 1126 Da, while sample STD (90:10) exhibited a M${}_{n}$ of 1189 (+63 Da). In contrast, for sample SYN (70:30) the M${}_{n}$ was 1104 Da, while sample STD (70:30) had M${}_{n}$ of 1168 (+63 Da). As these differences in both M${}_{n}$ and M${}_{w}$ can be attributed to many factors, such as the purity and nature of the termination fatty acids, the ratio between the fatty acids and acetic anhydride, the reaction temperature and time, the most plausible explanation is the effect of the non-oleic fraction (12%) in the samples which were prepared from olive waste. This is because all samples were prepared under the same reaction temperature and time, and thus, the only varying factors were the concentration of oleic acid (10, 30, 50, 70 wt%) and the source of it (standard grade and the one extracted from the waste).

### 3.4. Degradation and Percentage Mass Loss

**Figure 9:**
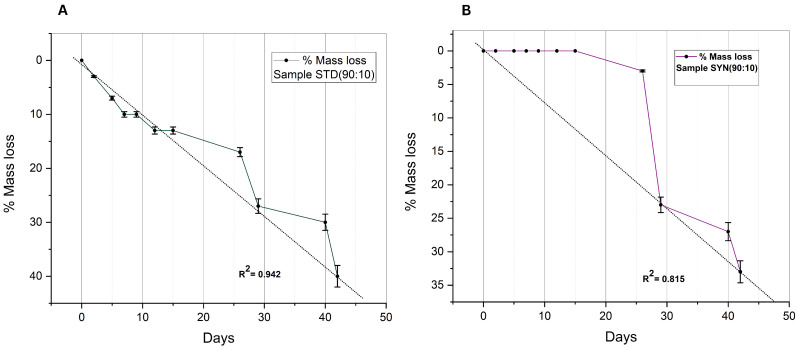
Degradation curves of samples: (**A**) STD (90:10); (**B**) SYN (90:10).

**Figure 10:**
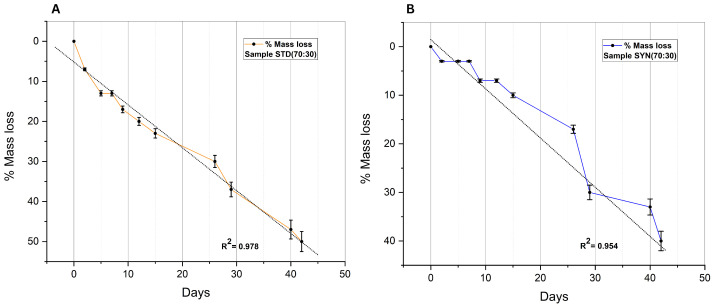
Degradation curves of samples: (**A**) STD (70:30); (**B**) SYN (70:30).

The rapid degradation of polyanhydrides due to their chemical structure can have detrimental effects on their properties. Ideally, the mass loss kinetics of a polyanhydride are limited to the surface of a polymer only, meaning to expect a surface erosion with linear mass loss. However, this does not validate that degradation can be strictly limited only to the surface as illustrated by numerous observations in which bulk eroding polymers degrade all over their cross section and have erosion kinetics that are non-linear and are usually characterized by a discontinuity [16]. The goal of copolymerizing and terminating polyanhydride materials with fatty acids, such as oleic acid, was to introduce a biocompatible and effective degrading polymer with a release rate that can be controlled by the hydrophobic properties of the fatty acid building blocks. Thus, the general hypothesis was based on the concept of increasing the hydrophobicity of the polymer by termination with fatty acids to eventually reduce the overall degradation rate [18]. This hypothesis suggested that the more hydrophobic the terminals, the more it can retard water from penetrating into the polymer matrix to cleave the anhydride bonds and hence reduce the degradation rate of the polymer. The previously reported data concluded this hypothesis after a comparison between the degradation rate of non-terminated poly (sebacic acid) (PSA) and (C14–C18) fatty-acids-terminated poly (sebacic acid). PSA lost about 70% of its initial weight after 6 days, and during this period, the fatty-acid-terminated polymers lost up to 20% of their initial weight. In line with this concept, it is logical to expect a decrease in the degradation rate with an increased weight ratio of the terminating fatty acid (oleic acid) or in other words, the polymer which copolymerized with more fatty acid ratio should have a longer degradation time due to the high resistance to water penetration. On the contrary, the mass loss data of the synthesized polyanhydrides revealed the opposite of this expectation. As seen from the degradation profiles, an adverse correlation between the degradation rate and the concentration of oleic acid was noticed in all samples. Sample STD (90:10), which has the lowest fatty acid concentration, lost about 40% of its mass at the end of the experiment, which was reduced to 33% in sample SYN (90:10), which was synthesized from olive waste. Sample STD (70:30) lost 50% of its mass; this was also reduced to 40% in sample SYN (70:30). The loss percentage continued to increase as the concentration of oleic acid increased. The fastest degradation profile was that of sample STD (30:70), which had the highest oleic concentration. This adverse effect of termination may be explained by the tendency of water molecules to expedite inward toward the anhydride bond and away from the hydrophobic terminals. The linearity of degradation curves was examined as indication for the surface erosion mechanism. The higher the proportion of the variance, the more linearly the sample was degraded as illustrated in Figure 9 and Figure 10 bellow and Figure S5. As shown in Figure 9B, the erosion curve of sample SYN (90:10) started with a constant induction period before it rapidly descended. This phenomenon was not observed on other samples which terminated by the standard oleic acid. The constant induction period indicates unchanged molecular weight and anhydride bond degradation due to zero water penetration rate; this perhaps can be explained by the increased crystalline regions of the sample, which in turn increased the resistance to water penetration and degradation more than amorphous regions.

The hydrolysis of polyanhydrides can be monitored by (a) weight loss of the sample, (b) disappearance of the anhydride bonds by IR spectroscopy, and (c) changes in polymer molecular weight as by GPC. Confirming the degradation of the samples by addressing the loss in anhydride bond using the FT-IR is crucial to compare the chemical structure before and after the degradation.

**Figure 11:**
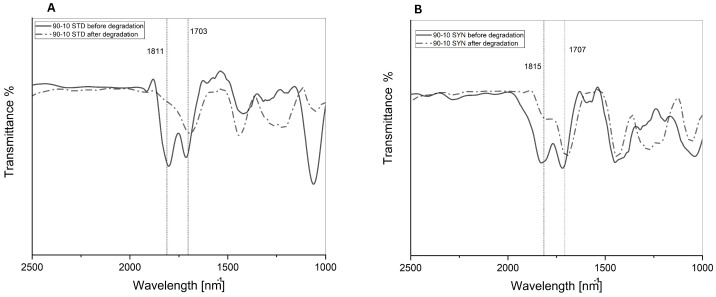
(**A**) Anhydride bond degradation in sample STD (90:10). (**B**) Anhydride bond degradation in sample SYN (90:10).

**Figure 12:**
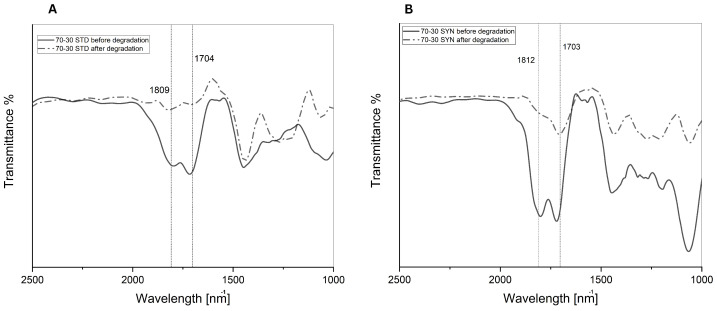
(**A**) Anhydride bond degradation in sample STD (70:30). (**B**) Anhydride bond degradation in sample SYN (70:30).

The % transmittance of the anhydride bond was diminished by 31% after degradation in sample SYN (90:10). In contrast, it diminished by 44.2% in sample STD (90:10) (Figure 11A,B). A higher percentage of anhydride bond loss was observed on the samples STD-SYN (70:30). For instance, sample STD (70:30) showed a 38% diminishing anhydride bond after degradation (Figure 12A,B). Sample SYN (70:30), which was copolymerized from waste oleic acid, exhibited the highest loss on anhydride bond by 70% diminishing transmittance. As seen from the data, both polymer weight loss percentage and anhydride bond loss confirmed the hydrolysis of the polymer matrices.

## 4. Conclusions

This work described the synthesis of terminated polyanhydrides with hydrophobic oleic acid, which were extracted and isolated from the solid waste of olive mills in Saudi Arabia by using microwave-assisted melt polycondensation. Polyanhydrides were terminated with varied oleic acid concentrations (10, 30, 50, and 70 wt%), and their chemical, molecular weights and degradation were characterized and analyzed. The size exclusion separation with urea crystallization was found to be effective to separate saturated and unsaturated fatty acids. It does not require excessive heat as do other methods, such as fractional distillation. Oleic acid was successfully separated from the mixture of waste pomace oil and reached a concentration of 88% without any chemical deterioration in the hydroxyl group or the double bond as confirmed by gas chromatography. Microwave-assisted polycondensation was performed using a conventional instrument with a rated power output of 900–1200 W. The reaction reached 160 °C within less than 2 min, and the reaction was carried on for 6–8 min with manual stirring. The chemical footprint of the synthesized polyanhydrides was confirmed by Fourier transform infrared spectroscopy (FT-IR). The data matched the literature regarding the anhydride bond stretch between 1740 and 1810 cm${}^{-1}$. Distinct bimodal stretch bands in the carbonyl region were observed, which is typical for polyanhydrides’ symmetrical and asymmetrical carbonyl stretching modes. The number average molecular weight (M${}_{n}$) and weight average molecular weight (M${}_{w}$) were determined by the GPC, and a close correlation was observed between the sebacic acid concentration and molecular weight in both number average molecular weight and weight average molecular weight. It was also noticed that varying the oleic acid concentration had no significant effect on either Mn and Mw in all the samples. The molecular weight of the samples was between 1300 and 1820 Da, which falls within the reported molecular weights of fatty-acids-terminated polyanhydrides, which are typically between 500 and 9000 Da. Degradation analysis was performed in phosphate buffer solution (pH = 7.0), and the weight loss percentage was measured for all samples over 42 days. Results showed that samples with the highest oleic acid concentration and lower molecular weights exhibited the highest rate of mass loss; on the other hand, samples with the lowest oleic acid concentration and highest molecular weights exhibited the lowest rate of mass loss at the end of the experiment. It was also observed that samples terminated with waste oleic acid tended to have lower degradation rates than samples terminated by standard-grade oleic acid. All samples showed a linear to semi-linear mass loss based on the fitting data of the degradation curves, which indicated the surface erosion mechanism. The degradation was also monitored by measuring the loss in the anhydride bond before and after the experiment using an FTIR instrument. A higher percentage of anhydride bond loss was observed on samples STD-SYN (70:30) by (38–70%) compared with samples SYN-STD (90:10) (31–44%).

## Data Availability

Not applicable.

## Supplementary Materials

The following supporting information can be downloaded at: https://www.mdpi.com/article/10.3390/polym14224799/s1, Figure S1: Gas chromatography of the initial fatty acid methyl ester sample (FAME2) before urea crystallization, Figure S2: Gas chromatography of sample (C2C1F1) with the highest oleic concentration after urea crystallization, Figure S3: FT-IR of samples SYN-STD (90:10), Figure S4: FT-IR of samples SYN-STD (70:30), Figure S5: Degradation curves of samples: (A) STD (50:50); (B) STD (30:70).
