# Supplementary material for: Preparation and Characterization of Polyanhydride Terminated with Oleic Acid Extracted from Olive Mills Waste
Source: Polymers (Basel). 2022 Nov 8;14(22):4799. doi: 10.3390/polym14224799 (PMC9698653; doi:10.3390/polym14224799)
Supplement: Supplementary file 1 [file polymers-14-04799-s001.zip › polymers-1997471-supplementary.pdf]

Preparation and characterization of polyanhydride terminated with oleic acid extracted from olive mills waste

Mustafa Zakiedin[1], Mansour Alhoshan[1, *], Maher Alrashed[1], Lahassen El Blidi[1].

[1] King Saud University, Department of Chemical Engineering, 11421-Riyadh, Saudi Arabia.

* Corresponding author: mhoshan@ksu.edu.sa

Supplementary Information

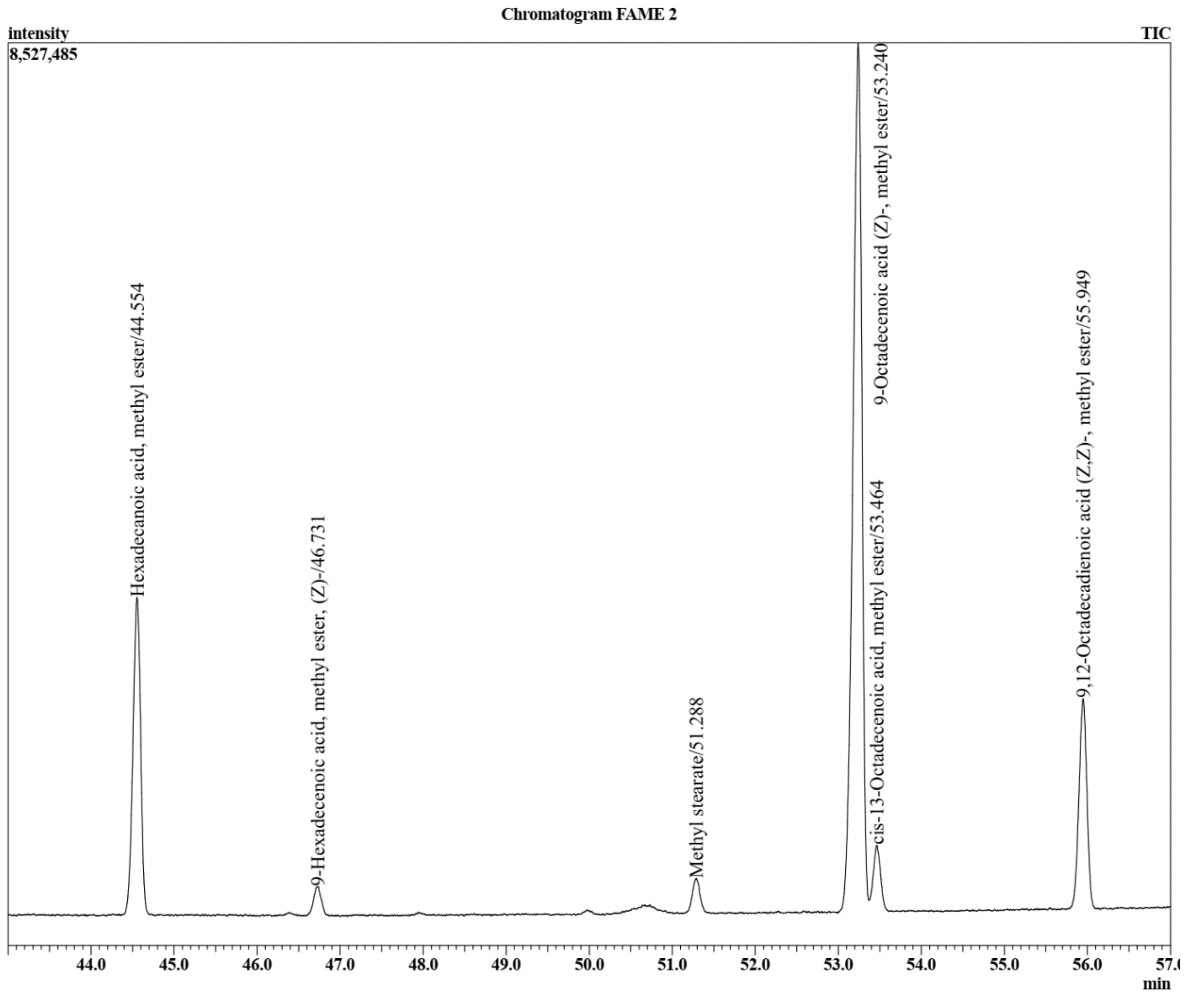

Figure S1 : Gas chromatography of the initial fatty acid methyl ester sample (FAME2) before urea crystallization.

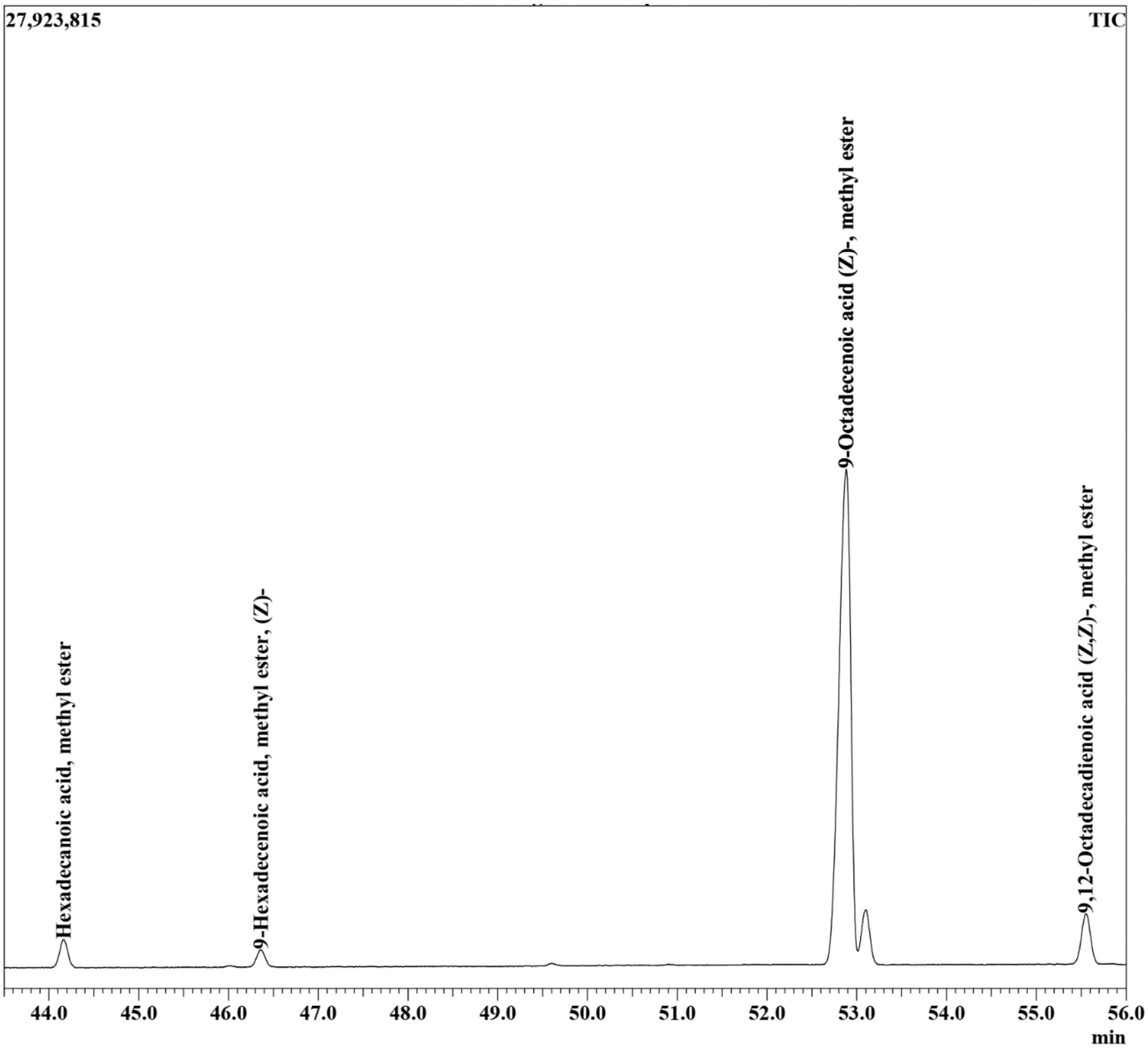

Figure S2 : Gas chromatography of sample (C2C1F1) with the highest oleic concentration after urea crystallization .

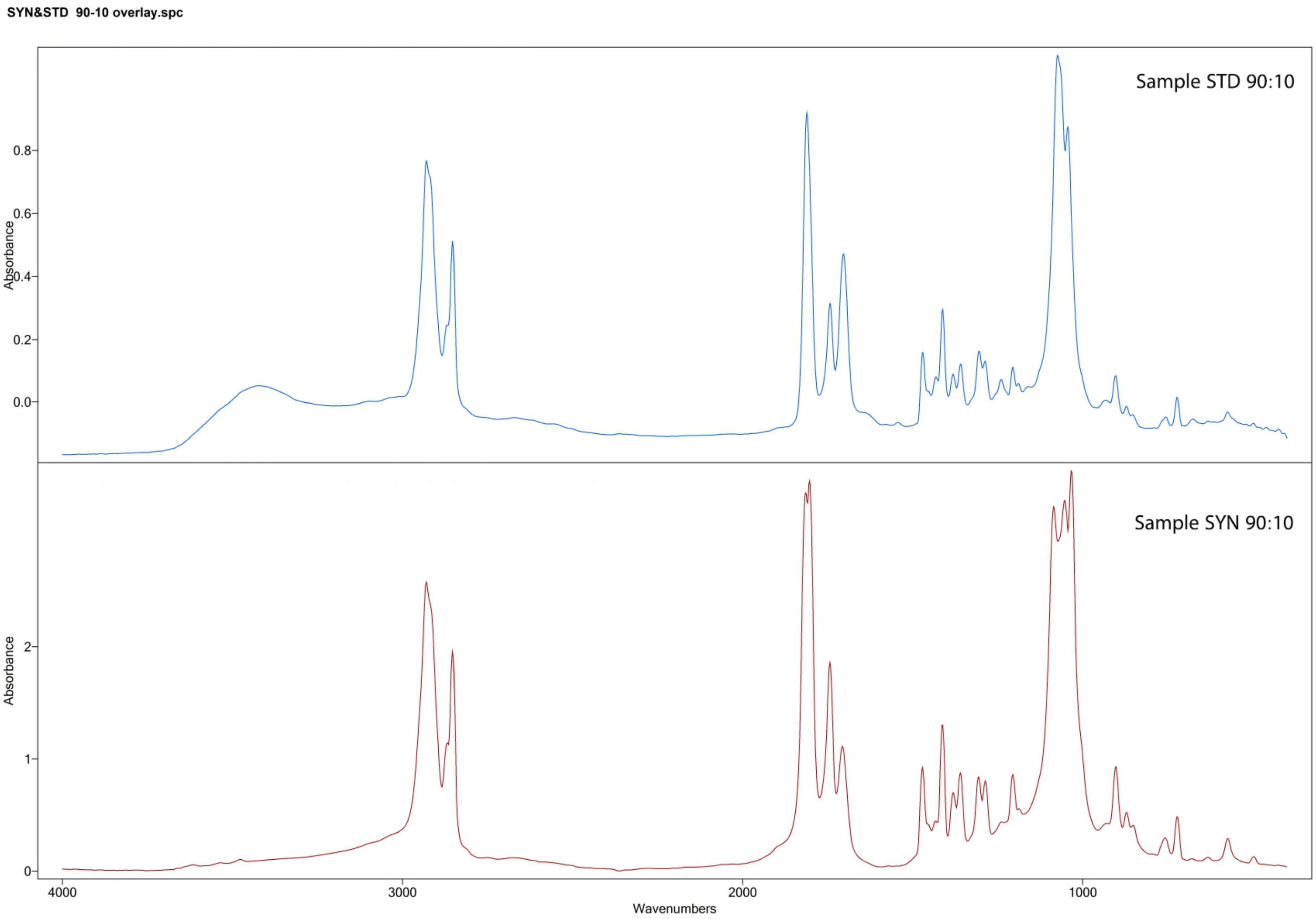

Figure S3 : FT-IR of samples SYN-STD (90:10).

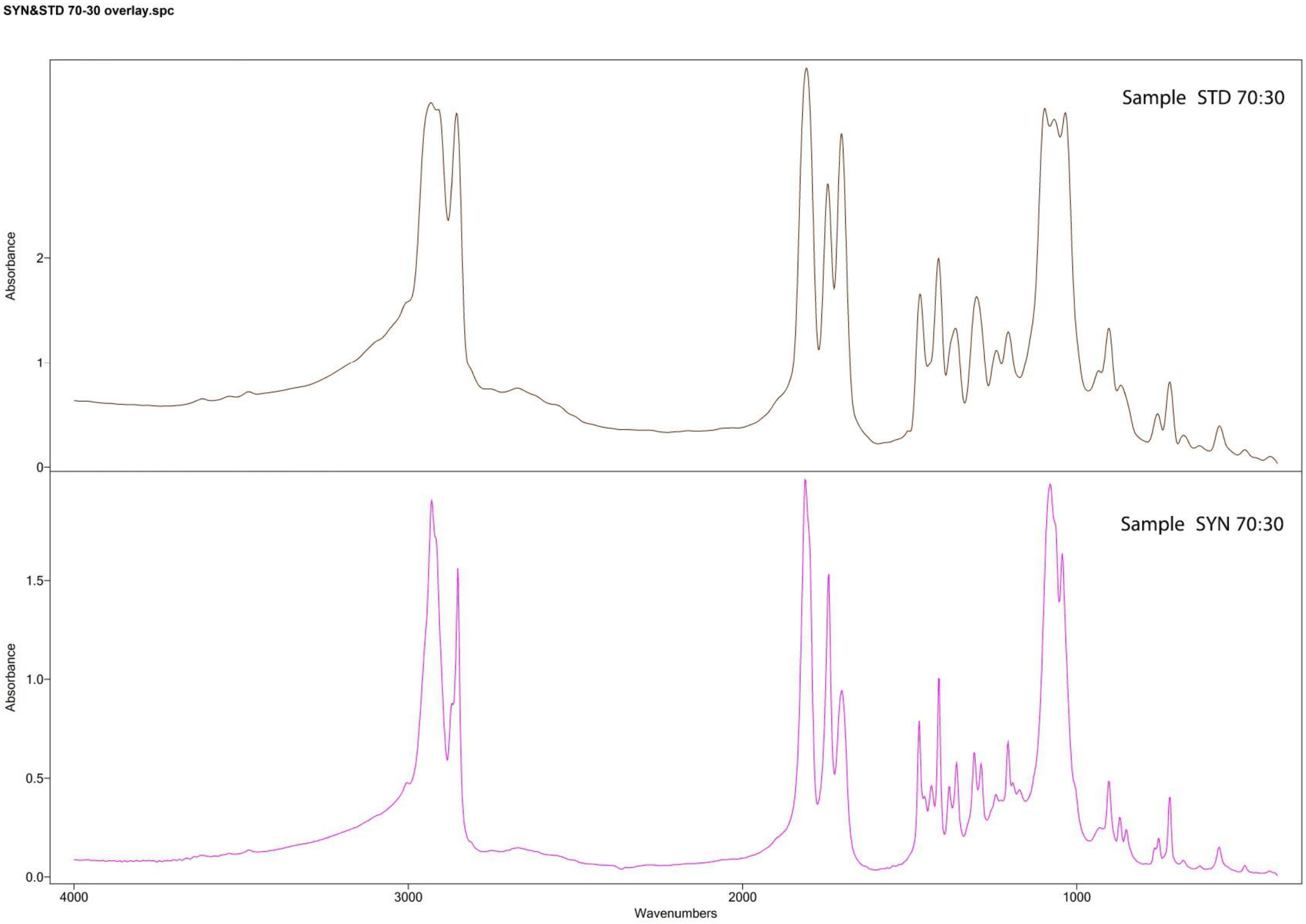

Figure S4 : FT-IR of samples SYN-STD (70:30).

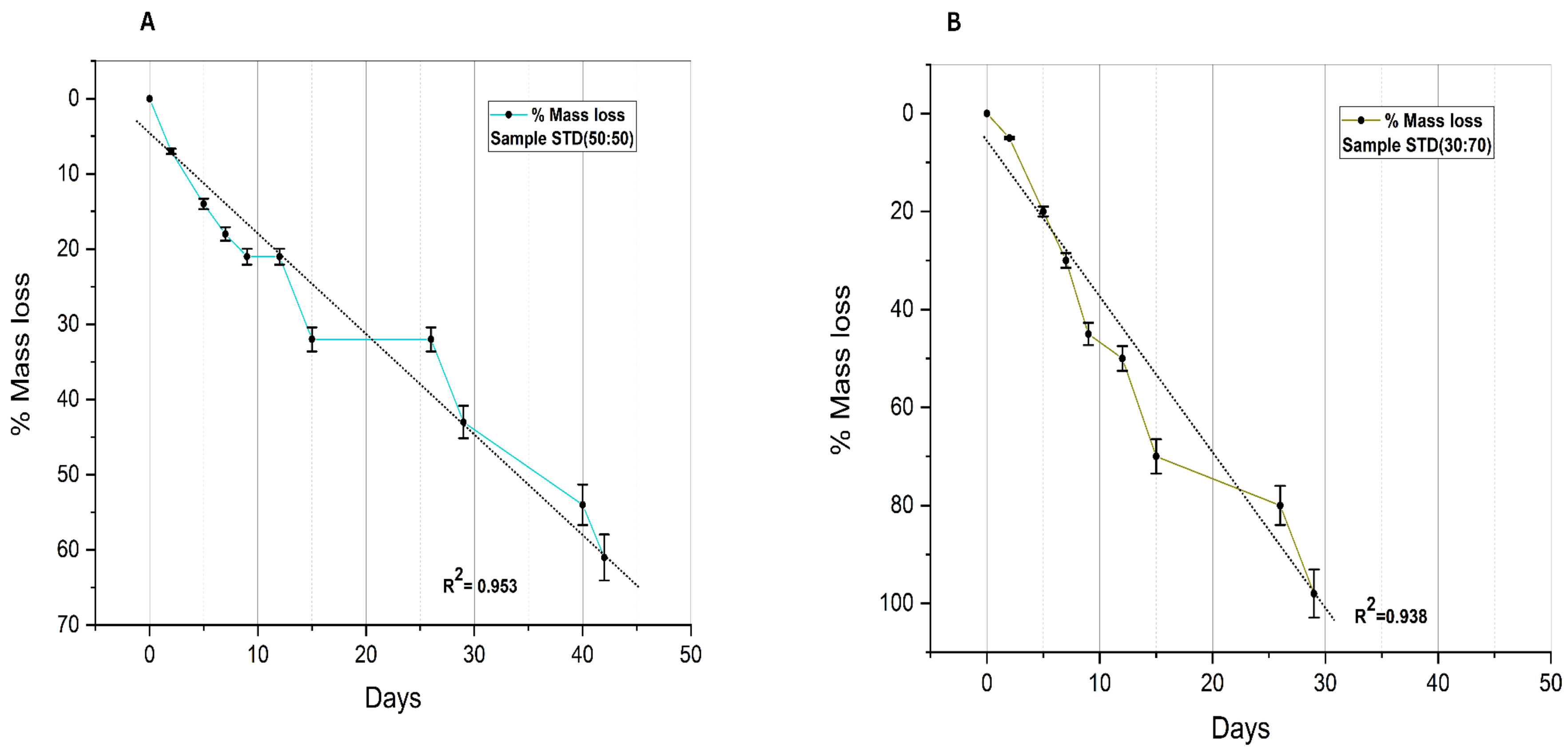

Figure S5: Degradation curves of samples: A) STD (50:50); B) STD (30:70).
